# Substrate Discrimination by ClpB and Hsp104

**DOI:** 10.3389/fmolb.2017.00036

**Published:** 2017-05-29

**Authors:** Danielle M. Johnston, Marika Miot, Joel R. Hoskins, Sue Wickner, Shannon M. Doyle

**Affiliations:** Laboratory of Molecular Biology, National Cancer Institute, National Institutes of HealthBethesda, MD, United States

**Keywords:** ClpB, Hsp104, molecular chaperone, disaggregase, DnaK, Hsp70, amyloid, aggregate

## Abstract

ClpB of *E. coli* and yeast Hsp104 are homologous molecular chaperones and members of the AAA+ (ATPases Associated with various cellular Activities) superfamily of ATPases. They are required for thermotolerance and function in disaggregation and reactivation of aggregated proteins that form during severe stress conditions. ClpB and Hsp104 collaborate with the DnaK or Hsp70 chaperone system, respectively, to dissolve protein aggregates both *in vivo* and *in vitro*. In yeast, the propagation of prions depends upon Hsp104. Since protein aggregation and amyloid formation are associated with many diseases, including neurodegenerative diseases and cancer, understanding how disaggregases function is important. In this study, we have explored the innate substrate preferences of ClpB and Hsp104 in the absence of the DnaK and Hsp70 chaperone system. The results suggest that substrate specificity is determined by nucleotide binding domain-1.

## Introduction

All cells have a protein network involved in maintaining the proteome following periods of stress. Maintenance of the proteome utilizes energy-dependent molecular machines that facilitate protein remodeling, reactivation, disaggregation and degradation of misfolded, aggregated or inactive proteins. Members of the Clp/Hsp100 family of ATP-dependent AAA+ proteins are molecular chaperones found in bacteria, archea, and the organelles of metazoans. Hsp104 and ClpB are two members of the Clp/Hsp100 family and are found in yeast and bacteria, respectively, where they are essential for growth following extreme stress, such as high temperature (Hodson et al., 2012; Doyle et al., 2013; Mogk et al., 2015). They aid in cell survival by disaggregating and reactivating proteins inactivated and aggregated following stress conditions (Hodson et al., 2012; Doyle et al., 2013; Mogk et al., 2015). Under normal growth conditions, Hsp104 and ClpB are not essential, however Hsp104, is required for the propagation of specific amyloidogenic proteins, prions, in yeast (Romanova and Chernoff, 2009; Tuite et al., 2011; Wickner et al., 2011; Winkler et al., 2012). Protein disaggregation and reactivation by Hsp104/ClpB require the collaboration of another molecular chaperone, Hsp70/DnaK and its cochaperones (Glover and Lindquist, 1998; Goloubinoff et al., 1999; Motohashi et al., 1999; Zolkiewski, 1999).

**Figure 1:**
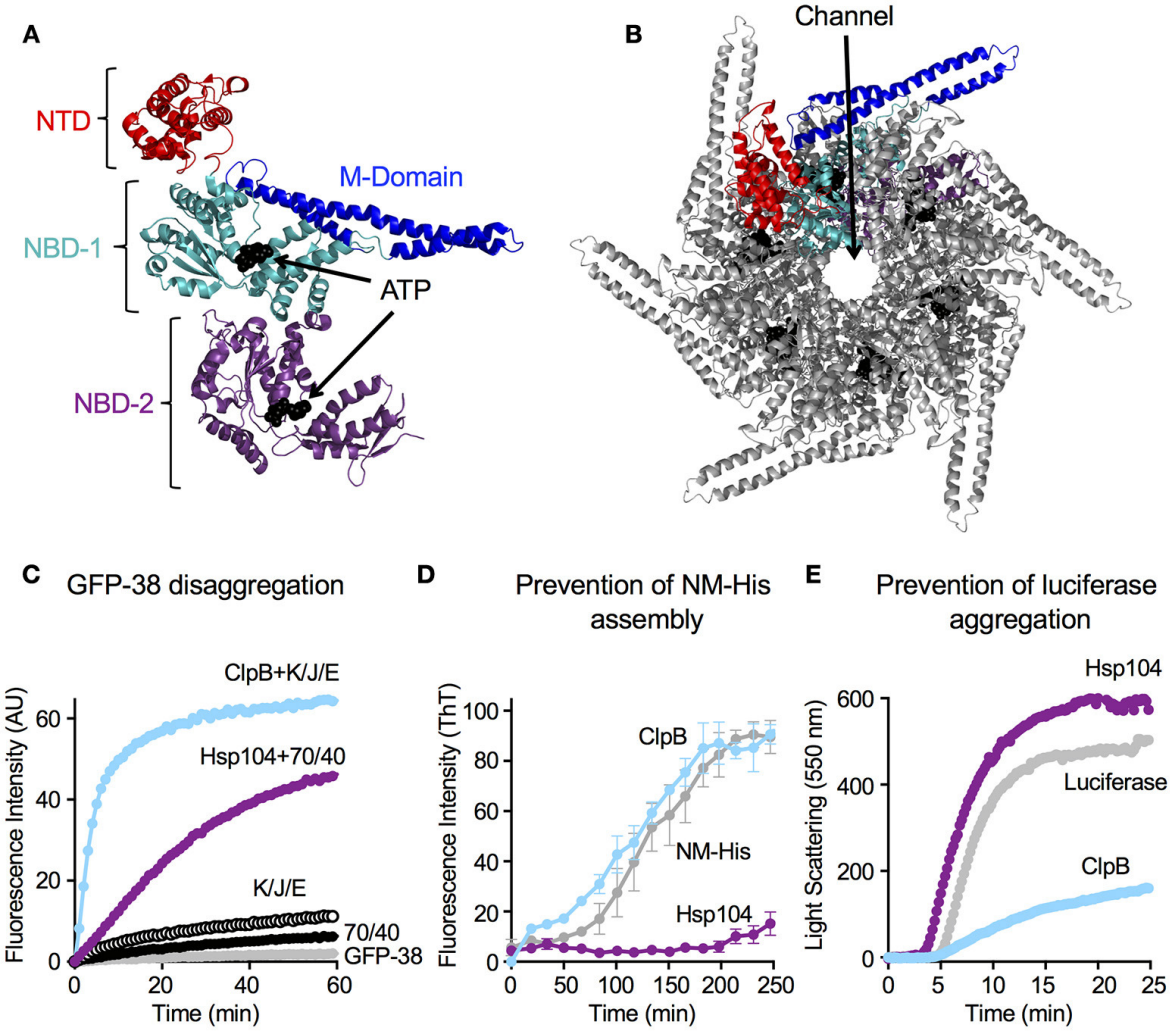
Hsp104 and ClpB have multiple chaperone activities *in vitro*. **(A)** Structure of the ClpB monomer from *Thermus thermophilus* bound to AMP-PNP (PDB code: 1QVR; chain C) is shown (Lee et al., 2003). Each monomer is comprised of an amino-terminal domain (N-domain; NTD; red), a coiled-coil middle domain (M-domain; blue) and two nucleotide-binding domains (NBD-1 and NBD-2; cyan and purple, respectively). The nucleotide is shown as a CPK model in black. **(B)** *T. thermophilus* ClpB hexamer model with bound ATP is shown (Lee et al., 2003; Diemand and Lupas, 2006). In **(B)**, one monomer of the hexamer is shown in color as described in **(A)**. **(C)** Hsp104 and ClpB can collaborate with the Hsp70 or DnaK system, respectively, in GFP-38 disaggregation, as observed by monitoring the increase in GFP fluorescence over time as described in Section Materials and Methods. **(D)** Hsp104, but not ClpB, can prevent the assembly of NM-His into amyloid fibers, as observed by monitoring Thioflavin T (ThT) fluorescence as described in Section Materials and Methods. Data are means ± *SEM* (*n* = 3). **(E)** ClpB, but not Hsp104, can prevent the aggregation of heat-denatured luciferase as observed by measuring turbidity by 90° light scattering as described in Section Materials and Methods. In **(C,E)** a representative experiment of three or more replicates is shown.

Hsp104 and ClpB, like other Clp/Hsp100 chaperones are hexameric ring-like structures (Diemand and Lupas, 2006; Erzberger and Berger, 2006; Doyle et al., 2013; Mogk et al., 2015). Recent studies have indicated that the Hsp104 hexamer may take on a spiral conformation at some point during the protein disaggregation process (Heuck et al., 2016; Yokom et al., 2016). Spirals have been observed previously when ClpA and ClpB were crystalized (Guo et al., 2002; Lee et al., 2003), however the importance of a spiral vs. closed ring architecture is not yet understood. Each protomer of the Hsp104/ClpB hexamer contains two highly conserved AAA+ modules, nucleotide binding domain-1 and -2 (NBD-1 and NBD-2), with each NBD possessing a Walker A and Walker B motif, an arginine finger motif and sensor-1 and -2 motifs (Hanson and Whiteheart, 2005; Erzberger and Berger, 2006; Wendler et al., 2012; Doyle et al., 2013; Figures 1A,B). The Hsp104/ClpB protomer also contains an N-terminal domain (N-domain, NTD), which is less conserved between species than the nucleotide binding domains. The NTD is connected to NBD-1 via a flexible linker and is important for interaction with some substrates (Lee et al., 2003; Nagy et al., 2010; Doyle et al., 2012; Zhang et al., 2012; Rosenzweig et al., 2015; Sweeny et al., 2015). Finally, a coiled-coil middle domain (M-domain), which is required for disaggregation activity, is inserted within NBD-1 of Hsp104/ClpB (Lee et al., 2003, 2007, 2010; Doyle et al., 2013; Mogk et al., 2015). The M-domain of Hsp104 and ClpB has been shown to directly interact with the Hsp70 chaperone, Ssa1 in yeast and DnaK in bacteria, in a species-specific manner (Sielaff and Tsai, 2010; Miot et al., 2011; Seyffer et al., 2012; Rosenzweig et al., 2013; Kummer et al., 2016). This direct interaction and collaboration is required for the synergy observed in ATP hydrolysis and substrate disaggregation (Doyle et al., 2007a; Miot et al., 2011; Seyffer et al., 2012; Rosenzweig et al., 2013; Kummer et al., 2016). Additionally, Hsp104 has a C-terminal domain that is involved in hexamerization and may play a role in thermotolerance (Mackay et al., 2008).

Although ClpB and Hsp104 require the DnaK/Hsp70 chaperone system for protein disaggregation *in vivo* and *in vitro*, alone they possess intrinsic protein remodeling activities: including protein unfolding, activation and disaggregation of small aggregates (Doyle et al., 2007b). The intrinsic chaperone activity is evoked by using mixtures of ATP and ATPγS or by using ATP hydrolysis defective ClpB/Hsp104 mutant proteins (Doyle et al., 2007b; Hoskins et al., 2009). One interpretation of the observations is that these conditions slow ATP hydrolysis by the chaperone allowing both substrate binding, a condition that requires ATP binding but not hydrolysis, and substrate translocation, a process that requires ATP hydrolysis, to occur simultaneously. By studying ClpB and Hsp104 using these conditions, ClpB and Hsp104 have been shown to function similarly to other Clp/Hsp100 chaperones. Briefly, Clp/Hsp100 chaperones recognize polypeptide substrates that contain an unstructured region of a minimum length, generally at an end. This unstructured region is engaged by residues in pore loops, which extend into the central channel of the Clp/Hsp100 hexamer (Baker and Sauer, 2012; Doyle et al., 2013; Mogk et al., 2015). These pore loops are in a nucleotide binding domain and use ATP driven conformational cycles to power mechanical unfolding of the polypeptide and translocation of the unfolded polypeptide through the channel (Weber-Ban et al., 1999; Lum et al., 2004, 2008; Schlieker et al., 2004; Siddiqui et al., 2004; Weibezahn et al., 2004; Hinnerwisch et al., 2005; Martin et al., 2008; Tessarz et al., 2008; Doyle et al., 2013). Unfolded substrate is then released and can refold spontaneously or with the help of additional chaperones (Hodson et al., 2012; Zolkiewski et al., 2012; Doyle et al., 2013; Mogk et al., 2015).

Substrate recognition and binding by Clp/Hsp100 chaperones, has been well-studied for many Clp proteins, including ClpA and ClpX, two bacterial chaperones associated with proteases (Weber-Ban et al., 1999; Zolkiewski, 2006; Baker and Sauer, 2012). Specific substrates have been identified by proteomic studies and specific recognition sequences have been determined (Flynn et al., 2003; Zolkiewski, 2006; Baker and Sauer, 2012). For ClpB and Hsp104 however, few specific substrates have been identified, and a mechanism for substrate discrimination by ClpB and Hsp104 has not been described. In the present study, we have further explored the question of substrate recognition by ClpB and Hsp104.

## Materials and methods

### Plasmids

pNM-His was constructed by amplifying the NM region of sup35 by PCR using 5′ and 3′ oligos containing Nde1 and BamHI sites, respectively, and pJC25NMstop (Addgene, plasmid #1228, Shorter and Lindquist, 2004) as template. The NM region PCR product does not contain a stop codon. This DNA was digested with Nde1 and BamHI and ligated into similarly digested pET24b. The resulting plasmid was digested with EcoR1 and Eag1 and a linker coding for six-histidines followed by a stop codon was ligated between the sites. The plasmid was confirmed by DNA sequencing.

### Purification of proteins

GroEL_Trap_ (Weber-Ban et al., 1999), Hsp104-ClpB chimeras (Miot et al., 2011), GFP-15 (Hoskins et al., 2002), GFP-38 and GFP-X_X_-H_6_ proteins (Hoskins and Wickner, 2006), and GFP (Hoskins et al., 2000) were purified as previously described. Luciferase was from Promega. Protein concentrations given are for monomeric GFP fusion proteins, NM-His and luciferase, hexameric ClpB, Hsp104 and chimeras, and tetradecameric GroEL_Trap_.

#### ClpB purification

ClpB wild-type and ClpB_E279A, E678A_ (Weibezahn et al., 2003; Doyle et al., 2007b) were constructed and purified as previously described (Zolkiewski, 1999), but with modifications. Cultures of *E. coli* BL21(DE3) containing pClpBwt (pET24b vector) or pClpB_E279A, E678A_ (pET24b vector) were grown at 30°C to OD_600_ of ~0.6 and then induced overnight with 0.1 mM IPTG. All purification steps were carried out at 4°C. Clarified cellular extracts were purified over a Q-Sepharose column (GE Healthcare) in 20 mM Tris-HCl, pH 7.5, 80 mM NaCl, 5 mM MgCl_2_, 0.5 mM EDTA, 20% glycerol (vol/vol) and 1 mM DTT. Proteins were eluted from the column with a linear gradient of 80–1,000 mM NaCl in the same buffer. Fractions containing ClpB were further purified using Sephacryl S-200 chromatography in 50 mM Tris-HCl, pH 7.5, 200 mM KCl, 10% glycerol, 20 mM MgCl_2_, 1 mM EDTA, and 1 mM DTT.

#### Hsp104 purification

Hsp104 wild-type and Hsp104_E285A, E687A_ (Bosl et al., 2005) were constructed and purified as previously described, but with minor modifications (Miot et al., 2011). This is a detailed description of our Hsp104 purification protocol. The plasmid pHsp104wt was used for the expression of tag-less, wild-type Hsp104 (Miot et al., 2011). pHsp104wt was transformed into *E. coli* strain Rosetta(DE3) by electroporation. Transformed cells were plated on LB plates supplemented with 50 μg/mL ampicillin and 10 μg/mL chloramphenicol and grown overnight at 32°C. Transformations were optimized to yield several hundred colony-forming units on each plate. The fresh transformants were used to inoculate Hsp104 expression cultures as follows: 5 mL of LB broth was added to each plate and the cells were resuspended using a sterile glass or plastic rod; cells from a single plate were used to inoculate 1 L of LB broth supplemented with 100 μg/mL carbenicillin (chloramphenicol was not added) in a 2 L baffled flask. Typically, 2–4 L of culture were grown at the same time for one preparation. The cultures were incubated with shaking at 25°C and 250 rpm to an OD_600_ = 0.25; the incubator temperature was reduced to 18°C and Hsp104 expression induced with the addition of IPTG to a final concentration of 0.1 mM; growth was continued overnight (14–16 h) at 18°C with shaking at 250 rpm. Cells were harvested by centrifugation in a pre-chilled rotor at 5,000 × g (~5,000 rpm in a Sorvall SLA-3000 or equivalent) for 10 min at 4°C. The cell pellet from each 1 L culture was resuspended in 25 mL ice cold Q104 buffer [40 mM Hepes pH 7.5, 80 mM NaCl, 0.5 mM EDTA, 20 mM MgCl_2_, 1 mM DTT, 20% glycerol (vol/vol), 5 mM ATP] containing EDTA-free complete protease inhibitor cocktail (Roche), which was prepared by mixing 1 tablet/50 mL of Q104 buffer. The resuspended cells were lysed by two or three passages through an ice-cold French Pressure cell (10,000 psi). The cell lysate was collected at the sample outlet tube with a vessel submerged in an ice bath. Cell debris was removed by centrifugation at 12,000 × g (10,000 rpm in a Sorvall SS-34 or equivalent) for 15 min at 4°C. The resulting supernatant was then centrifuged at 130,000 × g (35,000 rpm in a Sorvall F50L-8x39 or equivalent) for 30 min at 4°C. As an alternative, a single centrifugation step at 34,500 × g (17,000 rpm in a Sorvall SS-34 or equivalent) for 90 min at 4°C will produce similar results. The supernatant was used for subsequent purification. The supernatant must be subjected to the first column purification step without interruption or overnight storage or Hsp104 activity will be significantly reduced or lost. All purification steps were performed at 4°C using pre-chilled buffers. The clarified lysate was applied to a 20 mL Q-sepharose Fast Flow (GE Healthcare) column equilibrated with Q104 buffer at 1 mL/min using a peristaltic pump. The column was washed with two column volumes of Q104 buffer and protein was eluted with a 100 mL, 80–500 mM NaCl linear gradient in Q104 buffer. Column fractions of 3 mL each were collected. At this point, fractions containing Hsp104 can be stored at −80°C. Next, a 3 mL Q-sepharose Hsp104 peak fraction was applied onto a 40 mL Sephacryl S-200 High Resolution (GE Healthcare) column (1.5 cm I.D. × 30 cm length) equilibrated with SE104 buffer (20 mM Hepes pH 7.5, 200 mM KCl, 0.5 mM EDTA, 20 mM MgCl_2_, 1 mM DTT, 20% glycerol (vol/vol), 5 mM ATP) at 0.5 mL/min using a peristaltic pump. Fractions (1 mL) were collected and those containing purified Hsp104 were stored at −80°C. When thawed for use, individual fractions are divided into 100–200 μL aliquots and stored at -80°C to minimize the number of freeze-thaw cycles. Using this procedure, the Hsp104 activity is stable for at least 1 year.

#### NM-His purification

NM-His was purified as previously described (Glover et al., 1997) with modifications. Cultures (50–100 mL) of BL21(DE3) *clpP*- transformed with pNM-His were grown in LB (30 μg/mL Kan and 10 μg/mL Cam) at 37°C to an OD_600_ of ~0.6–0.8 and induced with 1 mM IPTG for 2 h. Cells were harvested by centrifugation and resuspended in 40 mM Hepes, pH 7.4, and lysed using a French Press. Urea was added to a final concentration of 8 M and the lysate kept at room temperature (~23°C) for the remaining preparation. Insoluble material was removed by centrifugation. NM-His was precipitated with the addition of MeOH to 70% (vol/vol) and the precipitate collected by centrifugation. The protein pellet was resuspended in NM Buffer (40 mM HEPES, pH 7.4, 8 M Urea) and then incubated with TALON resin for 30 min. The slurry was poured into an empty chromatography column and washed with 10 bed volumes of NM Buffer. NM-His was eluted with NM Buffer containing 50 mM Imidazole. NM-His containing fractions were precipitated with MeOH as above, the pellet was resuspended in 70% MeOH and the sample was stored at -80°C in small aliquots. NM-His was stable for ~6 months.

### GFP unfolding assay

Reaction mixtures (100 μL) contained buffer A [20 mM Tris-HCl, pH 7.5, 100 mM KCl, 5 mM DTT, 0.1 mM EDTA, and 10% glycerol (vol/vol)], 0.005% Triton X-100 (vol/vol), 0.2 mg/mL BSA, 10 mM MgCl_2_, 2 mM ATP, and 2 mM ATPγS (Roche), an ATP regenerating system (20 mM creatine phosphate and 6 μg creatine kinase), 0.4 μM GFP or GFP fusion protein, 3.0 μM GroEL_*Trap*_ and 1 μM ClpB or Hsp104. GroEL_*Trap*_ is a mutant form of GroEL that binds but does not release unfolded proteins and was included in the reactions to prevent the GFP fusion proteins from refolding (Weber-Ban et al., 1999). Unfolding was initiated with the addition of ATP, ATPγS, and MgCl_2_ and the change in fluorescence signal was monitored over time at 25°C using a Tecan Infinite M200Pro plate reader. Excitation and emission wavelengths were 395 and 510 nm, respectively. For K_M_ and V_max_ determinations, substrate concentrations were varied between 0.1 and 10 μM while keeping ClpB and Hsp104 concentrations constant at 1 μM. GroEL_Trap_ was varied between 1 and 5 μM depending on the substrate concentration. Unfolding rates were determined from the initial linear decrease in fluorescence intensities of the GFP fusion proteins. Michaelis-Menten analysis was performed using the non-linear regression analysis in Prism 7.0a for Mac OS X, GraphPad Software, La Jolla California USA (http://www.graphpad.com).

### Protein complexes

Reaction mixtures (100 μL) containing GFP-15, GFP-X_30_-H_6_, or GFP-X_7_-H_6_ (0.4 μM) with or without ClpB_E279A, E678A_ or Hsp104_E285A, E687A_ (2 μM) were incubated in buffer A, 0.005% Triton-X100, 5 mM ATP, and 10 mM MgCl_2_ for 45 min at room temperature. Reaction mixtures were fractionated on a Sephacryl S200 column (GE Healthcare) equilibrated with 20 mM Tris-HCl, pH 7.5, 100 mM NaCl, 20 mM KCl, 0.1 mM EDTA, 10% glycerol, 5 mM ATP, and 10 mM MgCl_2_ at room temperature. Fractions (100 μL) were collected and GFP fluorescence was measured in a Tecan Infinite M200Pro plate reader at 25°C as described above. The percentage of the GFP fusion protein signal that was shifted upon chaperone binding was determined by calculating the area under the shifted peak compared to the total area under all peaks. The elution profile of ClpB_E279A, E678A_ or Hsp104_E285A, E687A_ (2 μM) was determined in the absence of GFP fusion protein by measuring protein in each fraction using the Bradford assay.

### Prevention of heat-denatured luciferase aggregation

Luciferase (0.2 μM) was denatured at 42°C in Buffer B (50 mM Tris-HCl, pH 7.5, 150 mM KCl, 20 mM MgCl_2_, 2 mM DTT) with 5 mM ATPγS in the presence or absence of 0.5 μM ClpB or Hsp104 as previously described (Weibezahn et al., 2003). Aggregation of luciferase was monitored as an increase in sample turbidity by measuring 90° static light scattering on a PerkinElmer LS55 luminescence spectrometer with excitation and emission wavelengths each set to 550 nm.

### Prevention of NM-His fiber assembly

NM-His fiber assembly reactions (100 μL) were initiated by diluting denatured NM-His in 8 M urea (20 mM Tris-HCl, pH7.4) 100-fold to a final concentration of 0.2 μM with assembly buffer (40 mM Hepes-KOH, pH 7.4, 150 mM KCl, 1 mM DTT) in the presence or absence of 0.5 μM ClpB or Hsp104 (Shorter and Lindquist, 2004). Assembly reactions were agitated at 1,000 rpm and assembly of NM-His fibers was assessed by Thioflavin T (ThT) binding (100 μM final concentration). ThT fluorescence was read in a Tecan Infinite M200Pro plate reader at 25°C using excitation and emission wavelengths of 440 and 481 nm, respectively.

### GFP-38 disaggregation assay

GFP-38 disaggregation was performed as previously described (Miot et al., 2011). Reaction mixtures (100 μL) contained 25 mM Hepes, pH 7.5, 50 mM KCl, 0.1 mM EDTA, 5 mM DTT, 0.005% Triton-X-100 (vol/vol), 4 mM ATP, an ATP regenerating system (10 mM creatine phosphate and 3 μg creatine kinase), 10 mM MgCl_2_, 5 μL heat-aggregated GFP-38 (prepared by heating 75–100 μL of 14 μM GFP-38 for 15 min at 80°C in 0.2 mL PCR tubes; the heated luciferase was rapidly frozen on dry ice, thawed and used immediately), 0.5 μM ClpB, 1.3 μM DnaK, 0.2 μM DnaJ and 0.1 μM GrpE or 0.5 μM Hsp104, 1.3 μM human Hsp70 (HSPA1A) and 0.2 μM Ydj1. GFP fluorescence was monitored over time at 23°C using a Varian Cary Eclipse fluorescence spectrophotometer with a plate reader. Excitation and emission wavelengths were 395 and 510 nm, respectively. Reactivation was determined compared to a non-denatured GFP-38 control.

## Results

### Hsp104 and ClpB exhibit substrate preferences

In this work, we wanted to know if ClpB and Hsp104 differ in their innate substrate preferences. The experiments addressing this question were performed in the absence of the DnaK or Hsp70 chaperone so it would be possible to study the basic properties of the ClpB/Hsp104 machine and avoid the complication of substrate recognition by DnaK and Hsp70. However, it is known that *in vivo* and *in vitro* in the presence of ATP, both ClpB and Hsp104 require DnaK/Hsp70 to carry out protein disaggregation and reactivation (Glover and Lindquist, 1998; Goloubinoff et al., 1999; Motohashi et al., 1999; Zolkiewski, 1999; Doyle et al., 2013).

**Figure 2:**
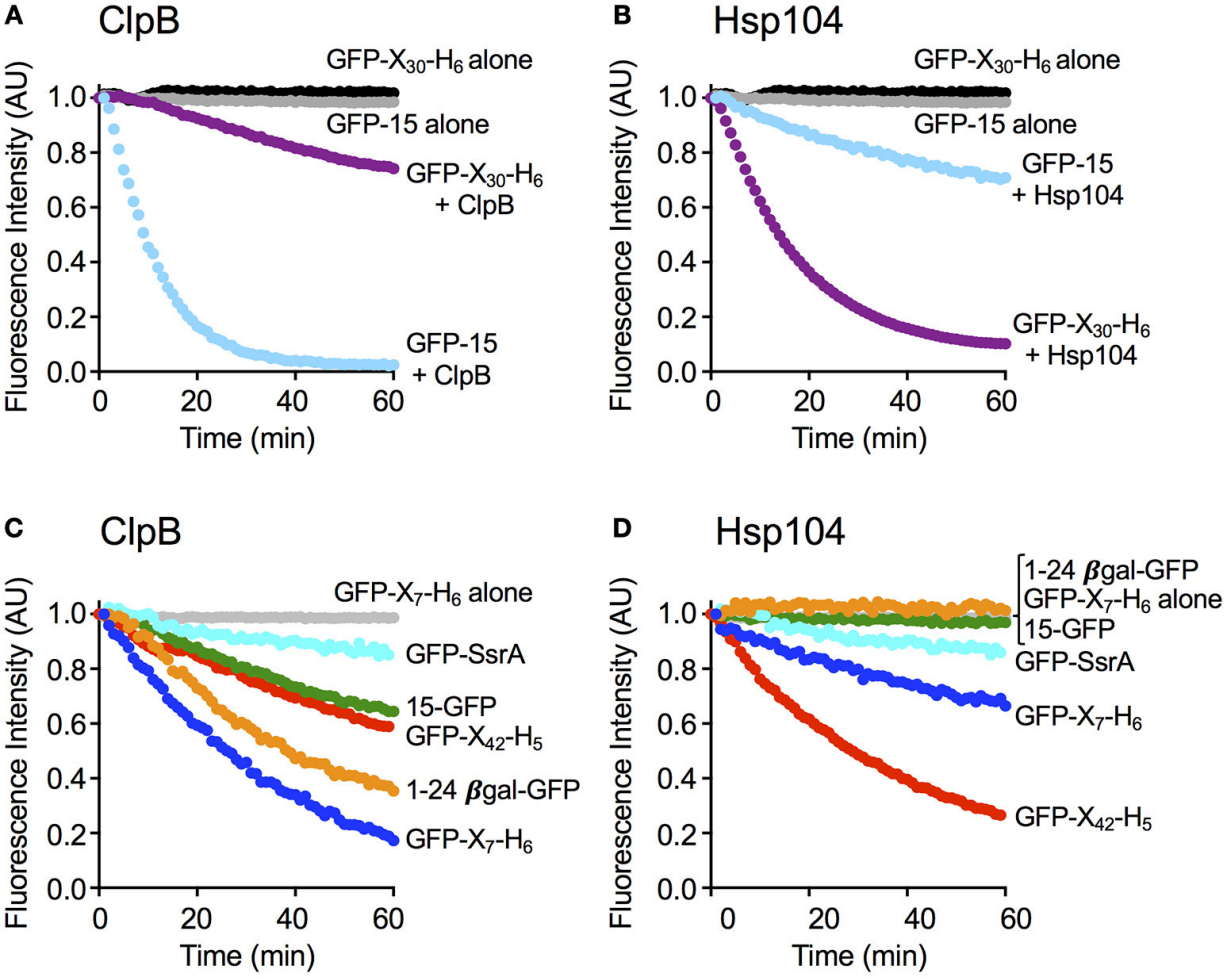
ClpB and Hsp104 exhibit specificity for substrate in unfolding reactions. **(A)** ClpB or **(B)** Hsp104 mediated unfolding of GFP-15 or GFP-X_30_-H_6_ in the presence of ATP and ATPγS as described in Section Materials and Methods. ClpB **(C)** or Hsp104 **(D)** mediated unfolding of additional GFP model substrates, GFP-SsrA, 15-GFP, GFP-X_42_-H_5_, 1–24 βgal-GFP, and GFP-X_7_-H_6_, in the presence of ATP and ATPγS. In **(A–D)**, the initial fluorescence was set equal to 1 and a data set representative of three or more replicates is shown. Substrates used in **(A–D)** are described in Table 1.

For these experiments, we used ClpB that was purified as described previously (Zolkiewski, 1999; Doyle et al., 2015; see Section Materials and Methods) and Hsp104 that was purified using standard biochemical protocols described in detail in Section Materials and Methods. The chaperones were isolated from *E. coli* cells overexpressing untagged ClpB or Hsp104 and consequently Hsp104 might not contain post translational modifications that would be present when the protein is expressed in yeast. Biochemical properties of Hsp104 were determined because controversy exists in the literature regarding several of the reported activities of Hsp104. Hsp104 isolated as described here reactivated aggregates in the presence of ATP in combination with Hsp70 and Hsp40 (Figure 1C; either yeast Ssa1 or human Hsp70 functioned in combination with Ydj1 or Sis1 from yeast; Miot et al., 2011; Reidy et al., 2014; Doyle et al., 2015). Additionally, it prevented amyloid assembly in the absence of ATP and Hsp70 (Figure 1D; Inoue et al., 2004; Shorter and Lindquist, 2004, 2006), and as previously observed it was unable to prevent aggregation of heat-denatured luciferase (Figure 1E; Glover and Lindquist, 1998). It also hydrolyzed ATP at a rate similar to published rates (Lum et al., 2004; Doyle et al., 2007b; Miot et al., 2011) and unfolded substrates using a condition that elicits the innate chaperone activity of Hsp104, a mixture of ATP and ATPγS (Figure 2; Doyle et al., 2007b). However, using Hsp104 prepared as described here, we were unable to repeat the observations, including one from our group, that Hsp104 accelerates assembly of the NM fragment of Sup35 in an ATP-dependent reaction (Shorter and Lindquist, 2004, 2006; Doyle et al., 2007b) and promotes disassembly of NM fibers in an ATP-dependent reaction in the absence of Hsp70 (Shorter and Lindquist, 2004, 2006; Doyle et al., 2007b; DeSantis et al., 2012). Other groups have previously reported that their Hsp104 preparations were unable to perform these two reported activities (Inoue et al., 2004; Krzewska and Melki, 2006; Savistchenko et al., 2008; Glover and Lum, 2009; Kummer et al., 2016).

To explore substrate discrimination by ClpB and Hsp104 in the absence of DnaK/Hsp70 we tested the two chaperones for the ability to act on several model substrates *in vitro*. The innate protein unfolding activity of ClpB and Hsp104 in the absence of the Hsp70/DnaK chaperone system was measured in the presence of a mixture of ATP and ATPγS to elicit the intrinsic chaperone activity (Doyle et al., 2007b, 2012; Hoskins et al., 2009). GFP-15, a GFP fusion protein containing a C-terminal 15-amino acid peptide was used as a model substrate. We had previously demonstrated that GFP-15 is a substrate for ClpA, but not ClpX (Hoskins et al., 2002), and we had also shown that ClpB catalyzes its unfolding in the presence of mixtures of ATP and ATPγS (Hoskins et al., 2009; Doyle et al., 2012; Table 1; Figure 2A). Unfolding of GFP-15 was determined by monitoring the decrease in GFP fluorescence over time in the presence of GroEL_Trap_, a mutant form of GroEL that binds and does not release unfolded proteins (Figure 2A; Weber-Ban et al., 1999). In contrast to the rapid rate of GFP-15 unfolding seen with ClpB, the rate of unfolding by Hsp104 was ~10-fold slower (Figure 2B). We next tested another GFP fusion protein that was previously shown to be a substrate for unfolding by ClpA, but not ClpX, GFP-X_30_-H_6_, which contains a C-terminal 30 amino acid peptide followed by a six-histidine tag (Hoskins and Wickner, 2006; Table 1; Figures 2A,B). ClpB unfolded GFP-X_30_-H_6_ at a much slower rate than it did GFP-15 (Figure 2A), however Hsp104 catalyzed unfolding of this substrate at a rate ~5-fold faster than ClpB (Figure 2B), showing that ClpB and Hsp104 differ in their ability to act on these substrates.

**Table 1:** GFP fusion proteins.

**Name**	**Tag location**	**Tag length**	**Tag sequence**
GFP-15^a^	C-terminus	15	MNQSFISDILYADIE
15-GFP	N-terminus	15	MNQSFISDILYADIE
GFP-SsrA	C-terminus	11	AANDENYALAA
1-24βGal-GFP^b^	N-terminus	24	MTMITDSLAVVLQRRDWENPGVTQ
GFP-X_7_-H_6_^c^	C-terminus	13	KLAAALEHHHHHH
GFP-X_30_-H_6_	C-terminus	36	AVHMASMTGGNNMGRDPNSSSVDKLAAALEHHHHHH
GFP-X_42_-H_5_	C-terminus	47	PMFAYSESDLIDAVHMASMTGGNNMGRDPNSSSVDKLAAALEHHHHH

a *The 15 amino acid tag on GFP-15 and 15-GFP comprises the first 15 N-terminal residues of the P1 plasmid replication initiator protein, RepA (Hoskins et al., 2002)*.

b *The 24 amino acid tag on 1-24βGal-GFP comprises the first 24 N-terminal residues of β-galactosidase (Hoskins et al., 2002)*.

c *Each (X) sequence of varying length, from 42 to 7 amino acids, comprises residues resulting from the translation of varying portions of the pET24b multicloning site (Hoskins and Wickner, 2006)*.

*GFP fusion proteins were constructed as described in Section Materials and Methods*.

We then wanted to know if ClpB and Hsp104 also differed in their ability to recognize and unfold GFP proteins with other polypeptide tags fused at either the N- or C-terminus. When two N-terminally tagged GFP fusion proteins, 15-GFP with the same 15 amino acid tag as on GFP-15 and 1-24βGal-GFP with a tag comprised of the first 24 amino acids of β-galactosidase, were tested, both substrates were unfolded by ClpB, as previously observed (Doyle et al., 2012; Table 1; Figure 2C). In contrast, neither of the N-terminally tagged substrates tested was detectably unfolded by Hsp104 (Figure 2D), supporting the above suggestion that ClpB and Hsp104 differ in their ability to unfold specific substrates. We next tested two additional C-terminally tagged GFP fusion proteins of different length but similar sequence, GFP-X_42_-H_5_ and GFP-X_7_-H_6_, which are related to GFP-X_30_-H_6_ (Table 1). Similar to the results observed for GFP-X_30_-H_6_, Hsp104 unfolded GFP-X_42_-H_5_ at a faster rate than ClpB (Figures 2C,D). However, GFP-X_7_-H_6_ was unfolded faster by ClpB than Hsp104, suggesting that Hsp104 may require a longer tag than ClpB, although the difference in unfolding rates may be due to sequence preferences or potential differences in the secondary structure of the tags (Figures 2C,D). We also tested GFP-SsrA, a GFP fusion protein C-terminally tagged with the well-studied SsrA 11-aa peptide, which can be recognized and unfolded by both ClpA and ClpX (Keiler et al., 1996; Singh et al., 2000; Table 1). Both ClpB and Hsp104 unfolded GFP-SsrA at a slow rate, indicating that the SsrA tag is poorly recognized by the two disaggregases (Figures 2C,D). This result is consistent with ClpB having weak binding affinity for the SsrA tag (Li et al., 2015) and observations previously reported, but not shown, indicating that ClpB does not unfold GFP-SsrA (Hinnerwisch et al., 2005). Taken together, ClpB and Hsp104 appear to have substrate preferences for protein unfolding.

**Figure 3:**
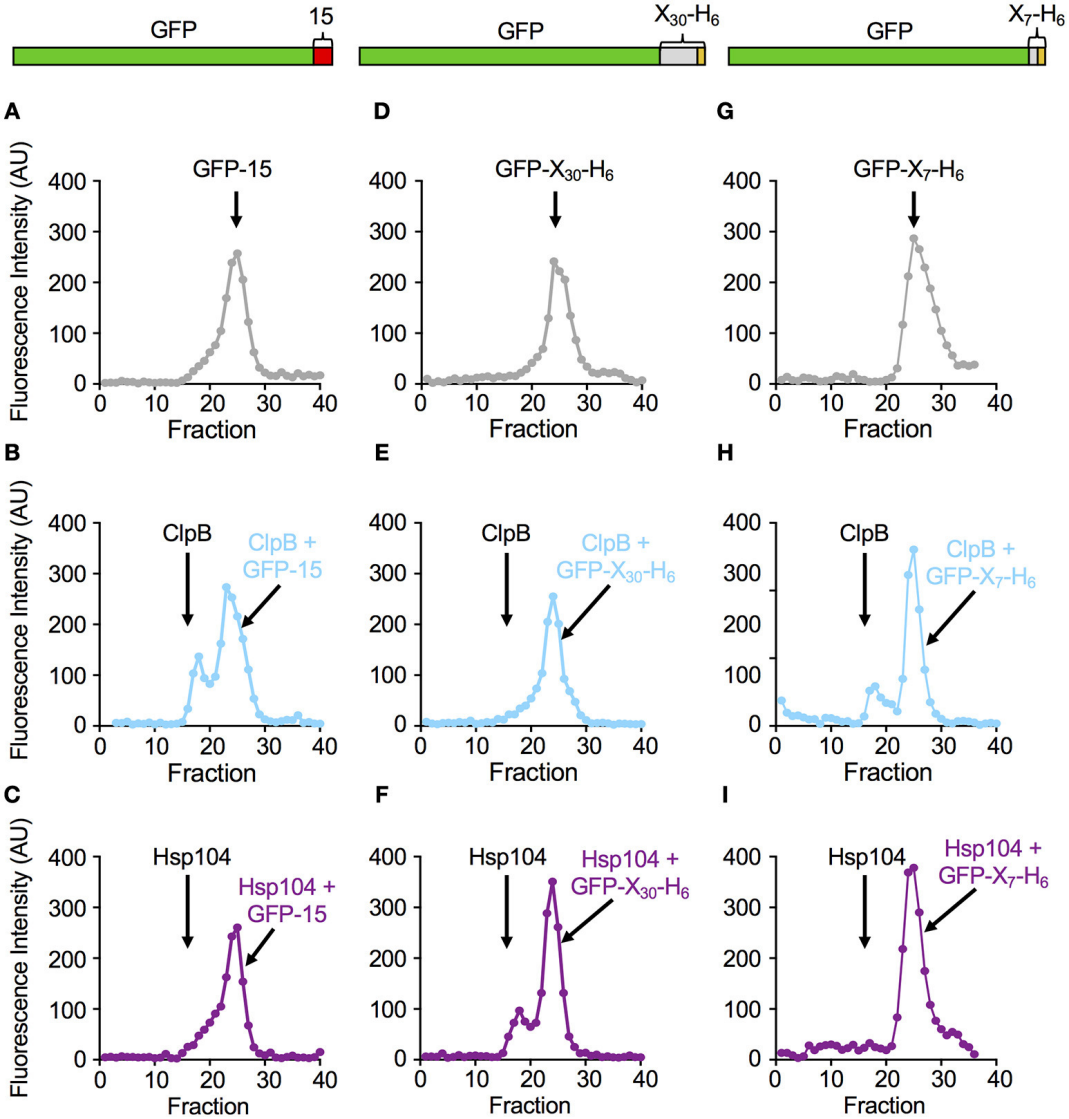
ClpB and Hsp104 substrate specificity is exhibited in complex formation. **(A–C)** Native GFP-15, **(D–F)** native GFP-X_30_-H_6_ or **(G–I)** native GFP-X_7_-H_6_ were incubated in the absence **(A,D,G)** or presence of ClpB_E279A, E678A_ **(B,E,H)** or Hsp104_E285A, E687A_ **(C,F,I)** in the presence of ATP as described in Section Materials and Methods. The position of the elution peak for ClpB alone **(B,E,H)** or Hsp104 alone **(C,F,I)** is indicated in their respective panels by an arrow. In **(A–I)**, each curve is a representative data set of three or more replicates.

We next tested if the rate of protein unfolding of a substrate correlated with the ability of the chaperone to interact stably with the specific substrate. Mutants of ClpB and Hsp104 with substitutions in the NBD-1 and NBD-2 Walker B sites (ClpB_E279A, E678A_ and Hsp104_E285A, E687A_) were used for these experiments because they bind but do not hydrolyze ATP and therefore limit the protein remodeling pathway to substrate interaction (Weibezahn et al., 2003; Bosl et al., 2005). ClpB_E279A, E678A_ was first incubated with GFP-15 in the presence of ATP to allow complex formation. Following incubation, the mixture was subjected to gel filtration chromatography and GFP fluorescence was measured in the eluted fractions. We observed a peak of fluorescence eluting near the position of ClpB_E279A, E678A_ and separated from the position where GFP-15 eluted when chromatographed alone (Figures 3A,B). About 27 ± 6% of the GFP-15 eluted in a complex with ClpB. However, when Hsp104_E285A, E687A_ was incubated with GFP-15 and the mixtures analyzed by gel filtration, there was no detectable peak of GFP-15 fluorescence eluting at the position of Hsp104_E285A, E687A_ (Figure 3C).

In parallel experiments, when ClpB_E279A, E678A_ was incubated with GFP-X_30_-H_6_ and ATP and analyzed by gel filtration, a single peak of GFP fluorescence was observed that eluted at the position of free GFP-X_30_-H_6_ (Figures 3D,E). In contrast, when Hsp104_E285A, E687A_ was incubated with GFP-X_30_-H_6_ and subjected to gel filtration, a peak of fluorescence, which contained 22 ± 2% of the total fluorescence, was detected eluting at the position of Hsp104 (Figure 3F). A third substrate, GFP-X_7_-H_6_ was also tested for its ability to interact with ClpB_E279A, E678A_ and Hsp104_E285A, E687A_ via gel filtration analysis (Figures 3G–I). The results were similar to those observed for GFP-15 with about 22 ± 1% of the GFP-X_7_-H_6_ eluting in a complex with ClpB (Figure 3H) while there was no detectable complex of GFP-X_7_-H_6_ and Hsp104 (Figure 3I). Thus, with these three substrates, the results indicate a direct correlation between the rate of substrate unfolding by ClpB and Hsp104 and the stability of substrate interaction by the chaperone.

**Figure 4:**
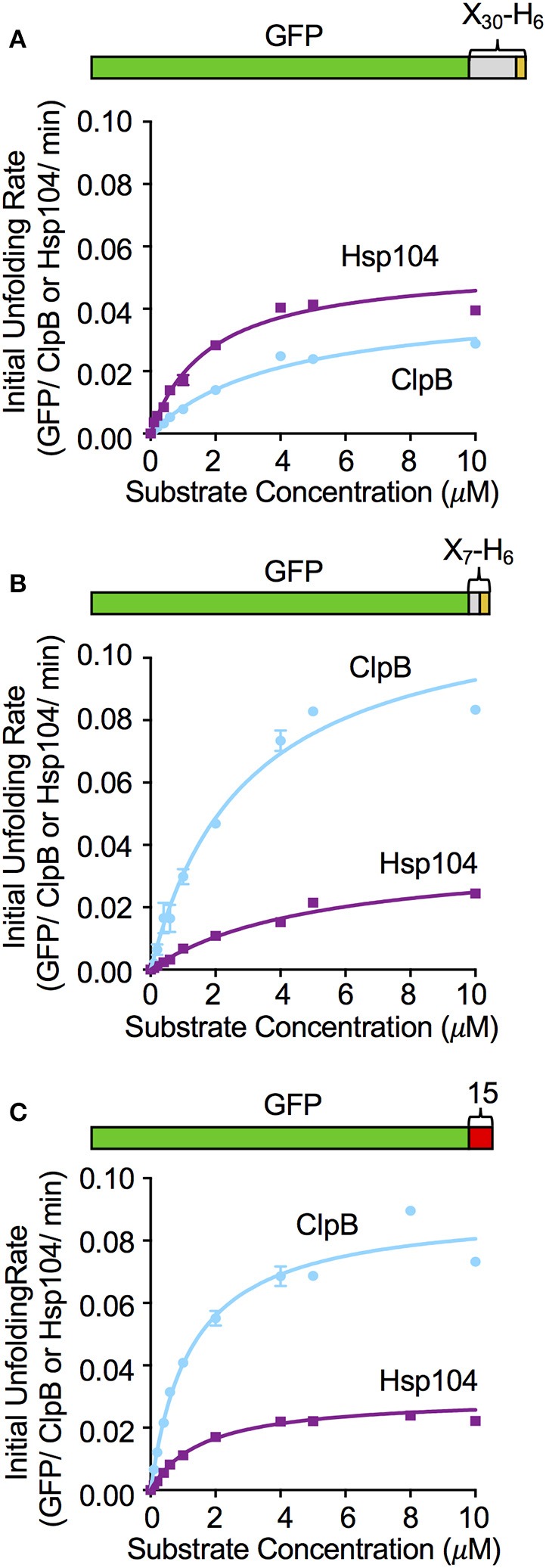
Effect of substrate concentration on the unfolding reaction by ClpB and Hsp104. The concentration of **(A)** GFP-X_30_-H_6_, **(B)** GFP-X_7_-H_6_, and **(C)** GFP-15 was varied in ClpB or Hsp104 mediated unfolding reactions and the initial rate of unfolding was plotted vs. the substrate concentration as described in Section Materials and Methods. Curves shown are the fit of the data to the Michealis-Menten equation and kinetic parameters (K_M_ and V_max_) were determined as described in the Section Materials and Methods. For GFP-X_30_-H_6_ **(A)** the Hsp104 K_*M*_ and V_max_ are 1.8 (0.2) μM and 0.054 (0.003) min^−1^, respectively, while the ClpB K_M_ and V_max_ are 4.1 (0.4) μM and 0.04 (0.002) min^−1^. For GFP-X_7_-H_6_ **(B)** the Hsp104 K_M_ and V_max_ are 5.2 (0.6) μM and 0.03 (0.001) min^−1^, respectively, while the ClpB K_M_ and V_max_ are 3.0 (0.4) μM and 0.12 (0.009) min^−1^. For GFP-15 **(C)**, the Hsp104 K_M_ and V_max_ are 1.6 (0.2) μM and 0.03 (0.001) min^−1^, respectively, while the ClpB K_M_ and V_max_ are 1.2 (0.1) μM and 0.091 (0.003) min^−1^. In **(A–C)**, data are the means ± *SEM* (*n* = 3).

To further investigate the relationship between the substrate binding affinity and the rate of substrate unfolding by ClpB and Hsp104, we monitored the initial rates of unfolding of GFP-X_30_-H_6_, GFP-X_7_-H_6_ and GFP-15, while keeping the chaperone concentration constant and varying the substrate concentration. For GFP-X_30_-H_6_, Michaelis-Menten analysis indicated that Hsp104 and ClpB similarly interact with this substrate (Figure 4A). Hsp104 only has an ~2-fold lower K_M_ and less than 2-fold higher V_max_ compared to ClpB. GFP-X_7_-H_6_ was bound similarly by Hsp104 and ClpB, with ClpB having less than a 2-fold lower K_M_ for binding than Hsp104 (Figure 4B). However, the maximum unfolding rate (V_max_) was ~4-fold higher for ClpB than for Hsp104 with this substrate (Figure 4B). When GFP-15 unfolding was analyzed in the same way, the K_M_ for both ClpB and Hsp104 was the same, however the maximum unfolding rates were again different, with ClpB having ~3-fold higher V_max_ than Hsp104 with this substrate (Figure 4C). These results indicate that for the substrates tested, binding affinity and the maximum substrate unfolding rate both affect the ability of ClpB and Hsp104 to efficiently process substrates.

### Nucleotide-binding domain-1 is important for determining substrate binding specificity

**Figure 5:**
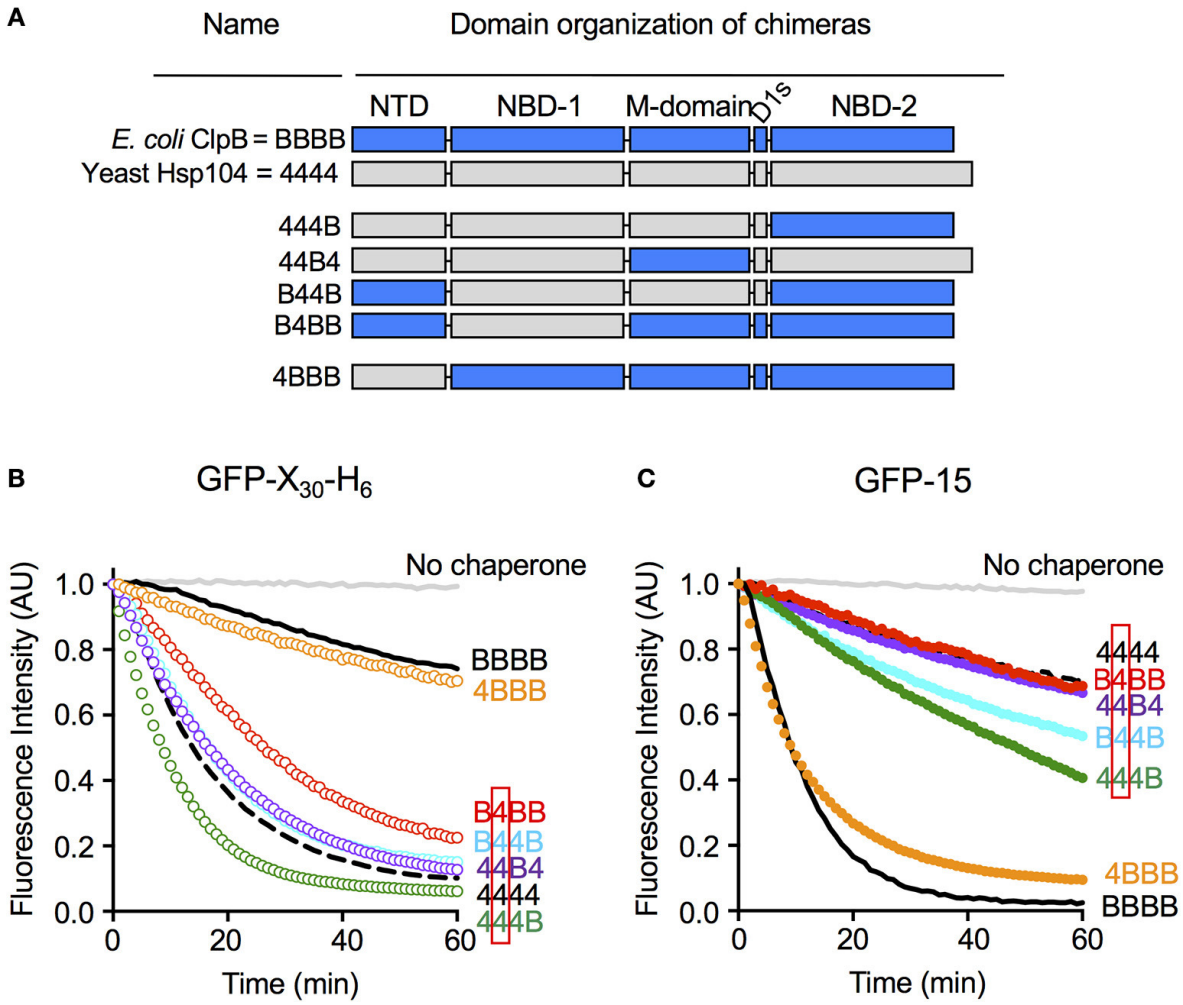
Nucleotide-binding domain-1 determines the specificity for substrate in unfolding reactions. **(A)** Domain organization of the ClpB-Hsp104 chimeras with domains from ClpB indicated by “B” and shown in blue, and domains derived from Hsp104 indicated by “4” and shown in gray. **(B,C)** Unfolding of GFP-X_30_-H_6_ **(B)** or GFP-15 **(C)** mediated by Hsp104 (4444; dashed black line), ClpB (BBBB; solid black line) or chimeras (colored lines) in the presence of ATP and ATPγS as described in the Section Materials and Methods. The initial fluorescence was set equal to 1 and a data set representative of three or more replicates is shown.

We wanted to explore substrate discrimination by ClpB and Hsp104 further by asking what domain or domains of ClpB and Hsp104 were involved in the substrate discrimination we observed with GFP-15 and GFP-X_30_-H_6_ (Figure 2). For these experiments, we utilized previously characterized chimeras of ClpB and Hsp104 (Miot et al., 2011). The chimeras are designated by a series of four characters that represent the four ClpB/Hsp104 domains from the N- to C-terminus, the N-domain, NBD-1, M-domain, and NBD-2 (Figure 5A). “B” represents a domain from ClpB and “4” represents a domain from Hsp104. For example, 444B represents the chimera with the N-domain, NBD-1 and M-domain from Hsp104 and NBD-2 from ClpB.

We tested the ClpB/Hsp104 chimeras for the ability to discriminate between GFP-X_30_-H_6_ and GFP-15, the two substrates most efficiently unfolded by Hsp104 and ClpB, respectively (Figures 2A,B). We observed that B4BB, a chimera with NBD-1 from Hsp104 and the other domains from ClpB, unfolded GFP-X_30_-H_6_ at a significantly faster rate than ClpB, although more slowly than Hsp104 wild-type (Figure 5B). This result suggests that the Hsp104 NBD-1 is important for substrate specificity. In support of this suggestion, three other chimeras containing the NBD-1 from Hsp104, B44B, 44B4, and 444B, also unfolded GFP-X_30_-H_6_ at rates similar to or slightly faster than Hsp104 wild-type (Figure 5B). Additionally, the observation that B44B unfolded GFP-X_30_-H_6_ like Hsp104 wild-type indicates that the N-terminal domain does not affect recognition of GFP-X_30_-H_6_ by Hsp104 (Figure 5B). 4BBB unfolded GFP-X_30_-H_6_ at a rate similar to ClpB wild-type, substantiating the conclusion that NBD-1 plays a role in substrate discrimination with this substrate, but the N-domain does not (Figure 5B).

We next monitored the ability of the chimeras to unfold GFP-15, the preferred substrate of ClpB (Figure 2A). As observed for GFP-X_30_-H_6_, chimeras with NBD-1 from Hsp104, including B4BB, B44B, 44B4, and 444B, functioned comparably to Hsp104 wild-type and unfolded GFP-15 at a slow rate (Figure 5C). The observation that B44B functioned like Hsp104 wild-type, again emphasized that the N-domain is not important for substrate specificity of this substrate (Figure 5C). Additionally, 4BBB, with the N-domain from Hsp104 and NBD-1 from ClpB, unfolded GFP-15 at a rate similar to ClpB wild-type (Figure 5C). Collectively, these results suggest that with the two substrates tested, NBD-1 is important for the substrate unfolding preference of Hsp104 and likely ClpB. Moreover, the N-domain does not appear to be involved in recognition of these substrates by ClpB and Hsp104.

## Discussion

In this study, we showed that Hsp104 and ClpB, in the absence of Hsp70 or DnaK, exhibit differing substrate preferences. By using chimeras of Hsp104 and ClpB domains we found that Hsp104 NBD-1 largely imparted the substrate specificity of Hsp104. The importance of NBD-1 in substrate binding and translocation has been demonstrated for many Clp/Hsp100 chaperones, including ClpX, ClpA, ClpB, and Hsp104, where it has been found that conserved tyrosines in the channel facing pore loops directly interact with substrates (Lum et al., 2004, 2008; Schlieker et al., 2004; Weibezahn et al., 2004; Hinnerwisch et al., 2005; Martin et al., 2008; Tessarz et al., 2008; Doyle et al., 2012). However, it is not clear what is uniquely different between NBD-1 of Hsp104 and NBD-1 of ClpB that is responsible for the substrate specificity that we observed. The NBD-1 pore loops of Hsp104 and ClpB are highly conserved suggesting additional residues in NBD-1 are potentially involved in substrate specificity. These additional substrate interactions may be with other residues in the central channel of NBD-1 or with residues in NBD-1 that are transiently exposed due to ATP-dependent conformational changes. Our results are consistent with a previous study by Tipton et al. that used chimeras of Hsp104 and ClpB to show that prion propagation in yeast requires NBD-1 from Hsp104 and that chimeras with ClpB NBD-1 were unable to support prion propagation (Tipton et al., 2008). Together, these results suggest that NBD-1 is important for substrate specificity of ClpB and Hsp104 in the absence of DnaK/Hsp70.

In our unfolding studies, we observed that ClpB and Hsp104 discriminate between GFP fusion proteins with different polypeptide tags fused at an end. Three of the substrates tested share almost the same 13 C-terminal residues, however, ClpB unfolded one (GFP-X_7_-H_6_) at a faster rate than Hsp104 while Hsp104 unfolded two (GFP-X_30_-H_6_ and GFP-X_42_-H_5_) faster than ClpB (Figure 2). These results suggest that either the length or the secondary structure of the recognition tag may affect the rate of substrate unfolding. In gel filtration studies monitoring substrate binding to ClpB and Hsp104, we observed a direct correlation between the rate of substrate unfolding by ClpB and Hsp104 and the stability of substrate interaction with the chaperone. However, Michaelis-Menten analysis of unfolding assays using three different substrates indicated there was only a 2-fold difference or less in binding affinities between Hsp104 and substrate or ClpB and substrate. The process of substrate unfolding is comprised of multiple steps including substrate recognition and binding, translocation and release, and the differences observed between Hsp104 and ClpB in substrate unfolding are likely due to more than just variances in sequence recognition. Additionally, the stability of the substrate and of the ClpB or Hsp104 hexamer are likely important for the overall substrate unfolding process.

The studies presented here using chimeras of Hsp104 and ClpB indicate that the N-domain of Hsp104 and ClpB does not affect the substrate discrimination observed with the two substrates tested. Previous studies addressed the role of the ClpB N-domain in substrate binding and unfolding and showed that the N-domain of ClpB is important for stabilizing ClpB and interaction with substrate (Nagy et al., 2010; Doyle et al., 2012; Rosenzweig et al., 2015). It was also shown that the N-domain directly interacts with substrates via a substrate-binding groove, and this interaction was nucleotide independent (Rosenzweig et al., 2015). Therefore, substrate interaction with the N-domain is different than the nucleotide-dependent binding observed between substrate and the NBD-1 pore loops (Schlieker et al., 2004; Weibezahn et al., 2004; Zolkiewski, 2006; Lum et al., 2008; Tessarz et al., 2008; Doyle et al., 2012; Rosenzweig et al., 2015). Additionally, previous work indicated that the N-domains may sterically obstruct access to the central channel and impede substrate binding to the pore loops of NBD-1 (Doyle et al., 2012; Nagy et al., 2010; Rosenzweig et al., 2015). In studies examining the role of the Hsp104 N-domain in protein unfolding and remodeling, it was observed that ΔN-Hsp104 was defective in substrate unfolding compared to Hsp104 wild-type, showing a role for the Hsp104 N-domain (Sweeny et al., 2015; Kummer et al., 2016). Therefore, for some substrates it is likely that the N-domain of ClpB/Hsp104 is required for stabilizing the initial interaction between chaperone and substrate and thus is required for the subsequent chaperone activity.

Understanding the mechanism of the intrinsic chaperone activity of ClpB/Hsp104 is providing the groundwork for understanding the more complex and biologically important reaction carried out by ClpB/Hsp104 in physical and functional collaboration with DnaK/Hsp70.

## Author contributions

DJ, MM, JH, SW, and SD designed the experiments. DJ, MM, JH, and SD performed the experiments. All authors were involved in data interpretation and discussion. SW and SD wrote the manuscript with contributions from JH.

### Conflict of interest statement

The authors declare that the research was conducted in the absence of any commercial or financial relationships that could be construed as a potential conflict of interest.
